# Supplementary material for: The intrinsically disordered protein TgIST from Toxoplasma gondii inhibits STAT1 signaling by blocking cofactor recruitment
Source: Nat Commun. 2022 Jul 13;13:4047. doi: 10.1038/s41467-022-31720-7 (PMC9279507; doi:10.1038/s41467-022-31720-7)
Supplement: Supplementary file 1 — Supplementary Information [file 41467_2022_31720_MOESM1_ESM.pdf]

Supplementary Information

The intrinsically disordered protein TgIST from Toxoplasma gondii inhibits STAT1 signaling by blocking cofactor recruitment

Zhou Huang[1], Hejun Liu[2]*, Jay Nix[3], Rui Xu[1], Catherine R. Knoverek[4], Gregory R. Bowman[4], Gaya K. Amarasinghe[2], L. David Sibley[1#]

Affiliations:

[1] Department of Molecular Microbiology, Washington University School of Medicine in St. Louis, St. Louis, MO 63110

[2] Department of Pathology and Immunology, Washington University School of Medicine, St. Louis, MO 63110, USA

[3] Molecular Biology Consortium, Advanced Light Source, Lawrence Berkeley National Laboratory, Berkeley, California 94720, USA.

[4] Department of Biochemistry and Molecular Biophysics, Washington University
School of Medicine, St. Louis, MO 63110, USA

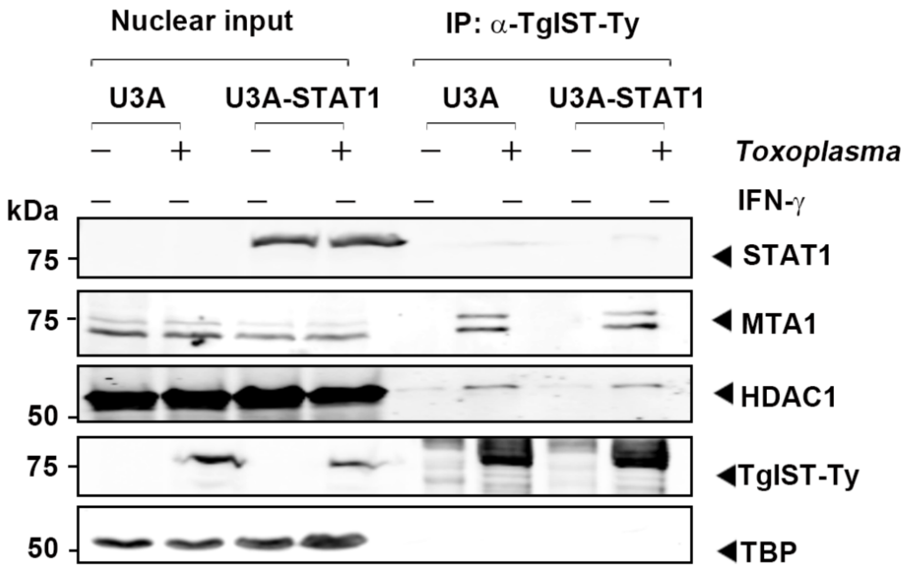

Supplementary Figure 1

The interaction between the secreted TgIST and STAT1 is IFN-γ dependent. Western blot analysis of host proteins following immunoprecipitation (IP) of TgIST-Ty from U3A (STAT1-null) or U3A-STAT1 (STAT1 complemented) cells that were infected with Toxoplasma (*vs*. mock) for 17 hr, all without IFN-γ treatment. Two core components of the Mi-2/NuRD complex, metastasis-associated protein (MTA1) and histone deacetylase 1 (HDAC1), were co-precipitated with TgIST-Ty. TATA-binding protein (TBP) was used as a negative control. Representative blots of two independent experiments with similar results were shown here.

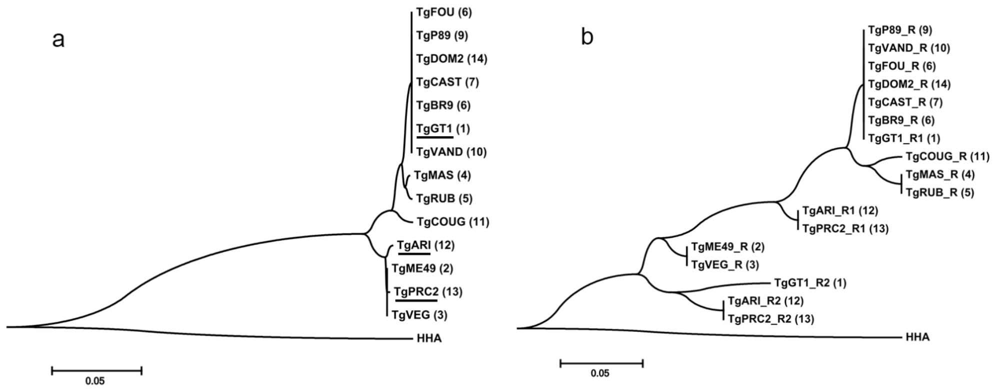

Supplementary Figure 2

Phylogenetic analysis ofTgIST from different lineages of *T.gondii*. (a) Neighbor-joining tree of full length TgIST from *T. gondii* strains (TG) or *Hammondia hammondi* (HHA). Strain types are indicated in the parentheses. Strains with a duplciated repeat region are underlined. (b) Neighbor-joining tree of individual repeat regions within TgIST from *T. gondii* strains (TG) or *Hammondia hammondi* (HHA). Protein sequences of TgIST were retrived from ToxoDB (https://www.toxodb.org/) and aligned using Muscle for mutiple sequence alignments (https://www.ebi.ac.uk/Tools/msa/muscle/). Phylogenetic trees were viualized by MEGA 7.0 (https://www.megasoftware.net/).

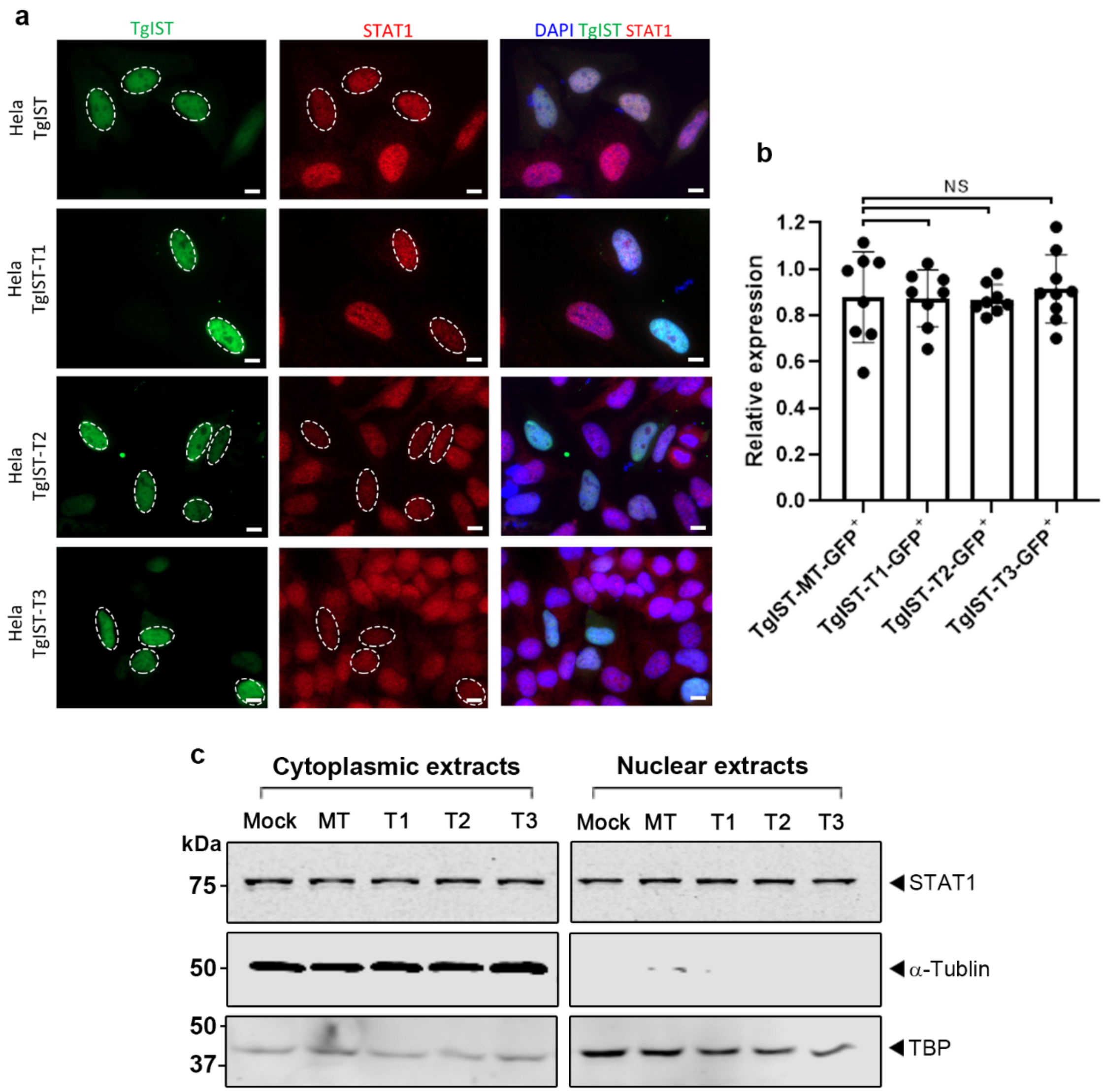

Supplementary Figure 3

STAT1 levels remain unchanged in TgIST expressing HeLa cells. (a) Representative images showing expression of STAT1 in transfected Hela cells. Hela cells transiently expressing GFP-tagged TgIST constructs for 24 hr were activated with IFN-γ for 6 hr followed by staining for GFP (Alexa Fluor 488, green), STAT1 (Alexa Fluor 568, red) and DAPI (blue). Scale bar = 10 µm. Representative micrographs of two independent experiments with similar results were shown here. (b) Quantification of STAT1 in TgIST-expressing (green, GFP^+) cells. The intensity of STAT1 were separately measured for GFP^+ and GFP^- cells, then normalized to GFP- cells. One-way ANOVA by Dunnett's test for multiple comparisons was used to compare expression divergency between samples. Data presented as mean + s.d. from two independent experiments. The top of the bar represents the mean, and the error bars represent the standard deviation. (c) Western blot analysis of cells lysates from transfected HeLa cells in (a). Equal amounts of total protein were separated by

SDS-PAGE, transferred to PVDF membranes, and incubated with corresponding primary antibodies as indicated. Blots for α-Tublin and TATA-binding protein (TBP) were used as controls for the cytoplasmic and nuclear fractions, respectively. Representative blots of two independent experiments with similar results were shown here.

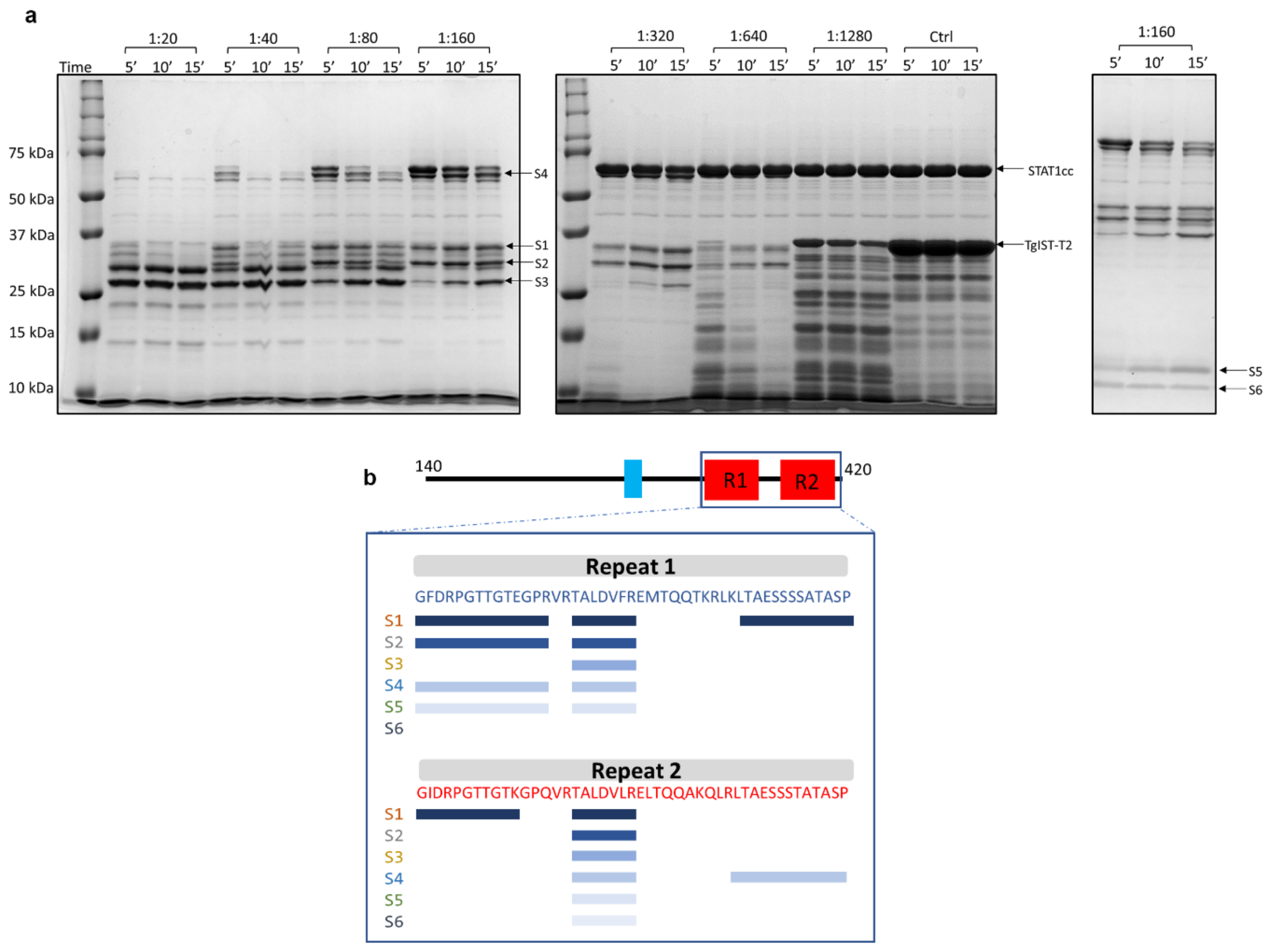

Supplementary Figure 4

Identification of the core STAT1 binding sequence in TgIST by limited trypsinization and mass spectrometery. (a) Purified TgIST-T2 complexed with STAT1cc was diluted to 10 µg in a 50 uL reaction volume. Dilutions of trypsin (1 mg/ml) from 1:20 to 1:1,280 (vol/vol %) were added to the TgIST-T2-STAT1cc complex and incubated for 5 min (5’), 10 min (10’) or 15 min (15’) as indicated. Reactions were stopped by addition of SDS sample buffer, followed by separation of samples by SDS-PAGE using 12% (the left and center gels) or 15% (the right gel) acrylamide gels. Resistant bands (numbered S1 – S6) from the samples treated with a 1:160 dilution of trypsin were cut from the gel and subjected to MS/MS analysis. Representative gel image of two independent experiments with similar results were shown here. (b) Limited proteolysis and mass spectrometry (MS) analysis identified core regions in the repeats of TgIST that were protected by interaction with STAT1. Purified TgIST-T2 complexed with STAT1cc was treated with trypsin and resistant bands were isolated from SDS-PAGE gels for MS analysis. Regions identified from MS

are shown as rectangles below the amino acids sequence of each repeat. S1 through S6 refer to partial degradation patterns detected by SDS-PAGE.

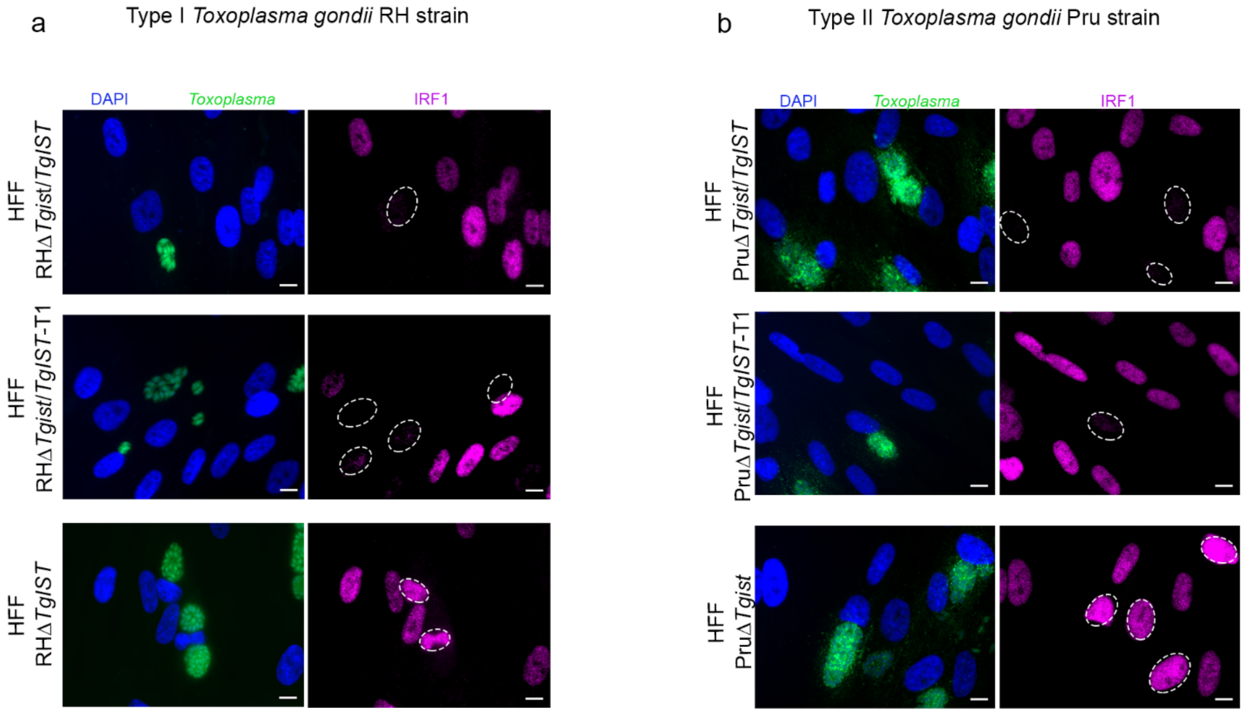

Supplementary Figure 5

Repression of IRF1 by TgIST is independent of Mi-2/NuRD interaction. Cells infected with type I (a) and type II (b) strains of *T. gondii*. HFF cells were plated on cover slips, infected for 6 hr with TgIST disruptant Type I (RH) or Type II (Pru) parasites, or strain complemented with wildtype TgIST (RH or PruΔ*Tgist*/*TgIST*) or TgIST-T1 (RH or PruΔ*Tgist*/*TgIST*-T1, lacks Mi-2/NuRD binding domain in its C-terminus). Cells were stimulated with IFN-γ (100 U/ml) for the last 18 hr, followed by staninning with α-IRF1. Scale bar = 10 μm. Representative micrographs of two independent experiments with similar results were shown here.

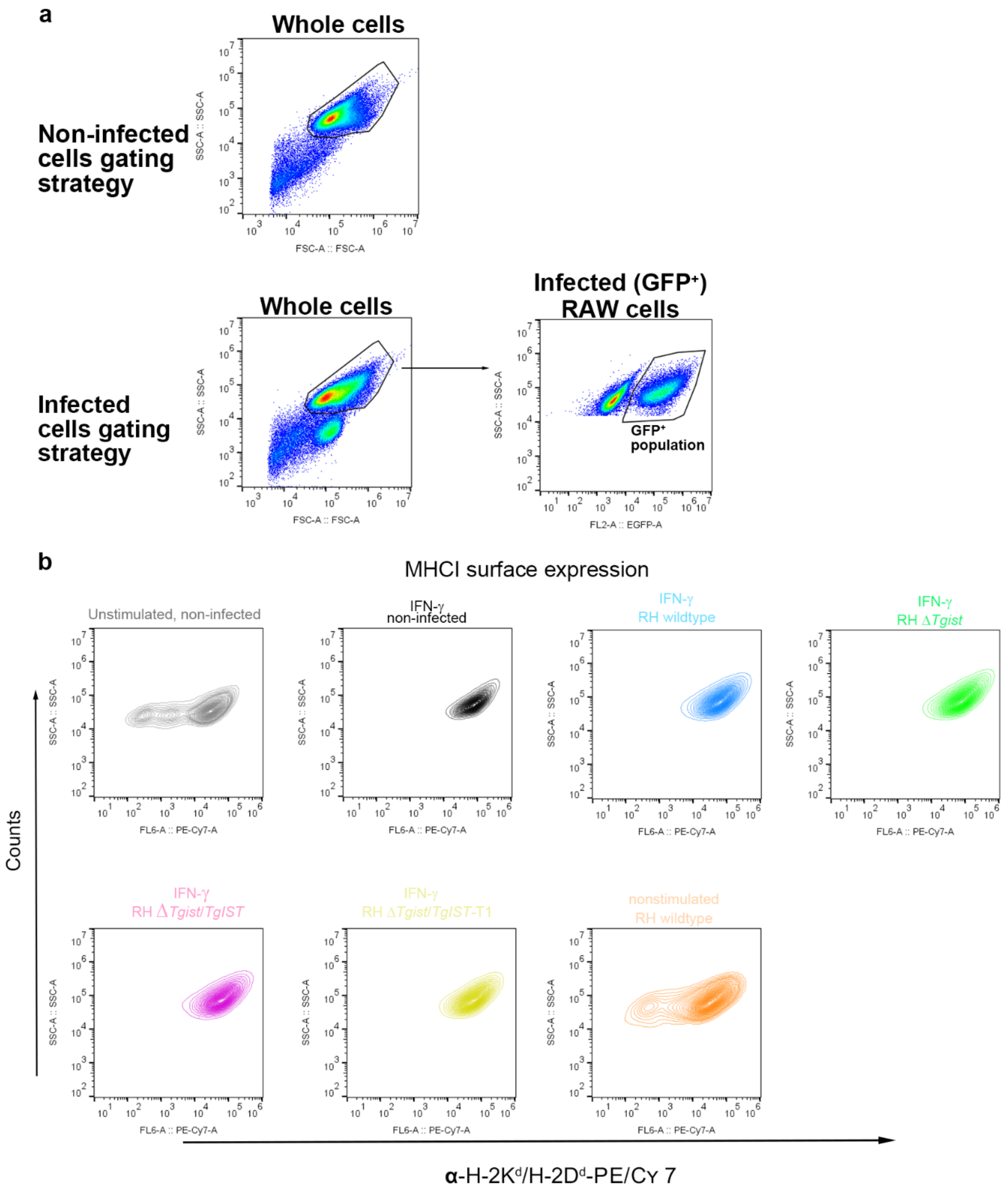

Supplementary Figure 6

Surface expression measurement of MHC I molecules on infected RAW246.7 cells. (a) Gating strategy of non-infected or *T. gondii* infected RAW246.7 cells used by flow cytometry experiments. For non-infected sample (the first panel in Fig 3a), cells were selected out from debris by FSC/SSC plot. For *Toxoplasma* infected sample (the rest panels in Fig 3a), whole cells were further gated by GFP+ populations, based on

harboring transgenic GFP-expression parasites. Representative of three independent experiments is shown. (b) TgIST can still block expression of STAT1 signalling without Mi-2/NuRD chromotin modification domain.

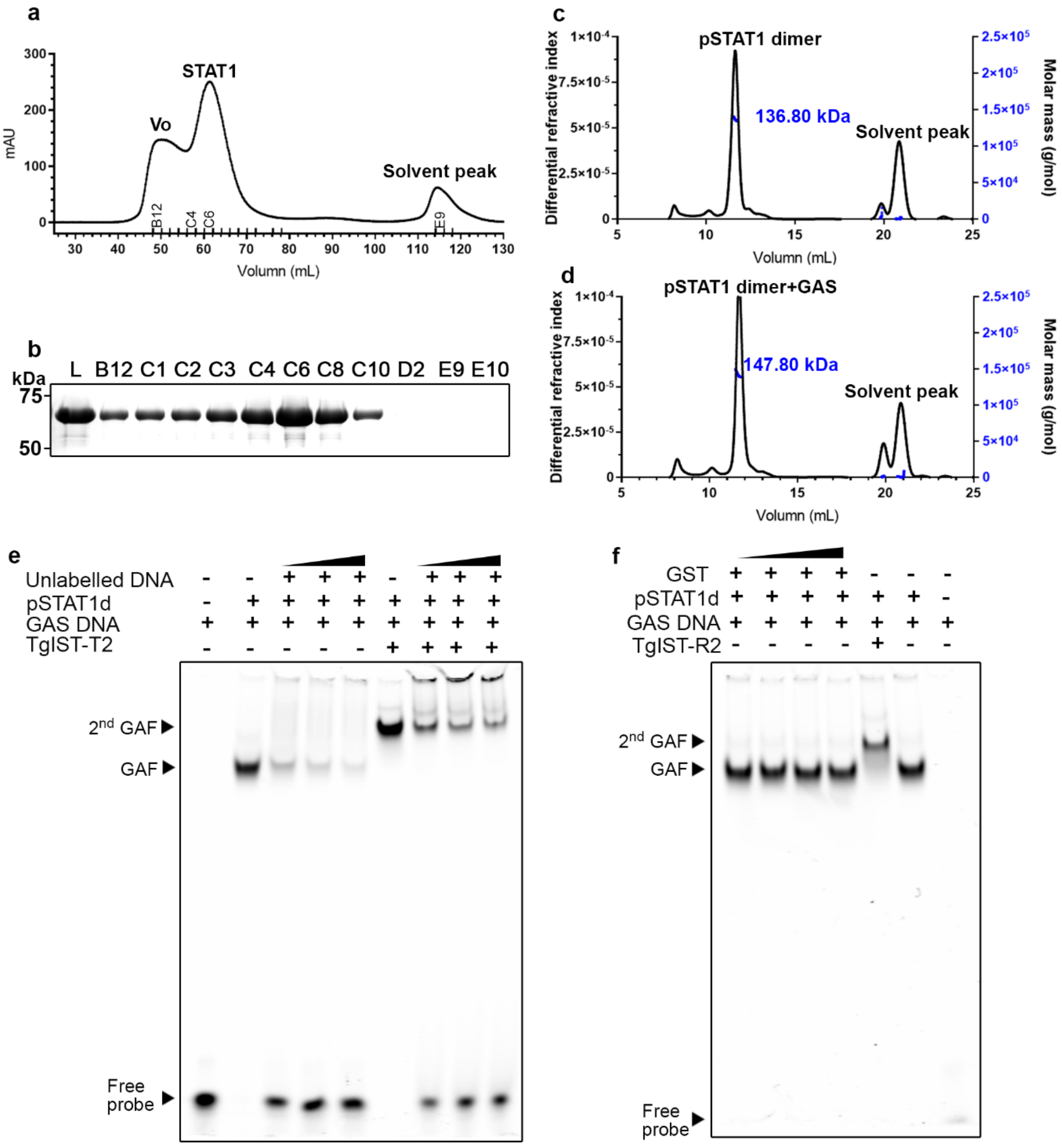

Supplementary Figure 7

Purification of phosphorylated STAT1 dimer and specificity controls for EMSA assays. (a) N-terminal Strep-tagged STAT1 was expressed in *E.coli* TKB1 cells that co-express ELK kinase in order to generate phosphorylated STAT1 dimers (pSTAT1d). pSTAT1d was successfully separated from STAT1 aggregates (Vo peak) by size exclusion chromatography. (b) SDS-PAGE analysis of column fractions from a. L, sample loaded on column. Representative blot of three independent experiments with similar results were shown

here. (c) Pooled fractions (C5-C10) (b) were concentrated and analyzed by multiple angle laser light scattering with in-line size exclusion chromatography (SEC-MALS).(d) Fractions C4-C10 (b) were pooled, mixed with a double stranded oligo for the GAS sequence, and subjected to SEC-MALS analysis. The observed molecular weights of pSTAT1d alone *vs*. bound to the GAS oligo were 136.8 and 147.8 kDa as measured by SEC-MALS, which compare favorably to their theoretical molecular weights of 137.0 kDa and 146.80 kDa, respectively. (e) EMSA experiments testing the specificity of formation of complexes in the absence or presence of TgIST. Addition of increasing concentrations of unlabeled DNA competitor reduced the formation of GAF in the absence of TgIST-T2 and the 2nd GAF in the presence of TgIST-T2. GAF, gamma-activated factor, 2nd GAF, super shifted form of GAS. (f) Addition of GST protein alone, which was used as the affinity tag in purification of TgIST-R2 in EMSA experiments shown in Figure 4, did not super shift the GAF complex. Black triangles indicate increasing concentration of components added based on label at the top. Representative gel images (e and f) of two independent experiments with similar results were shown here.

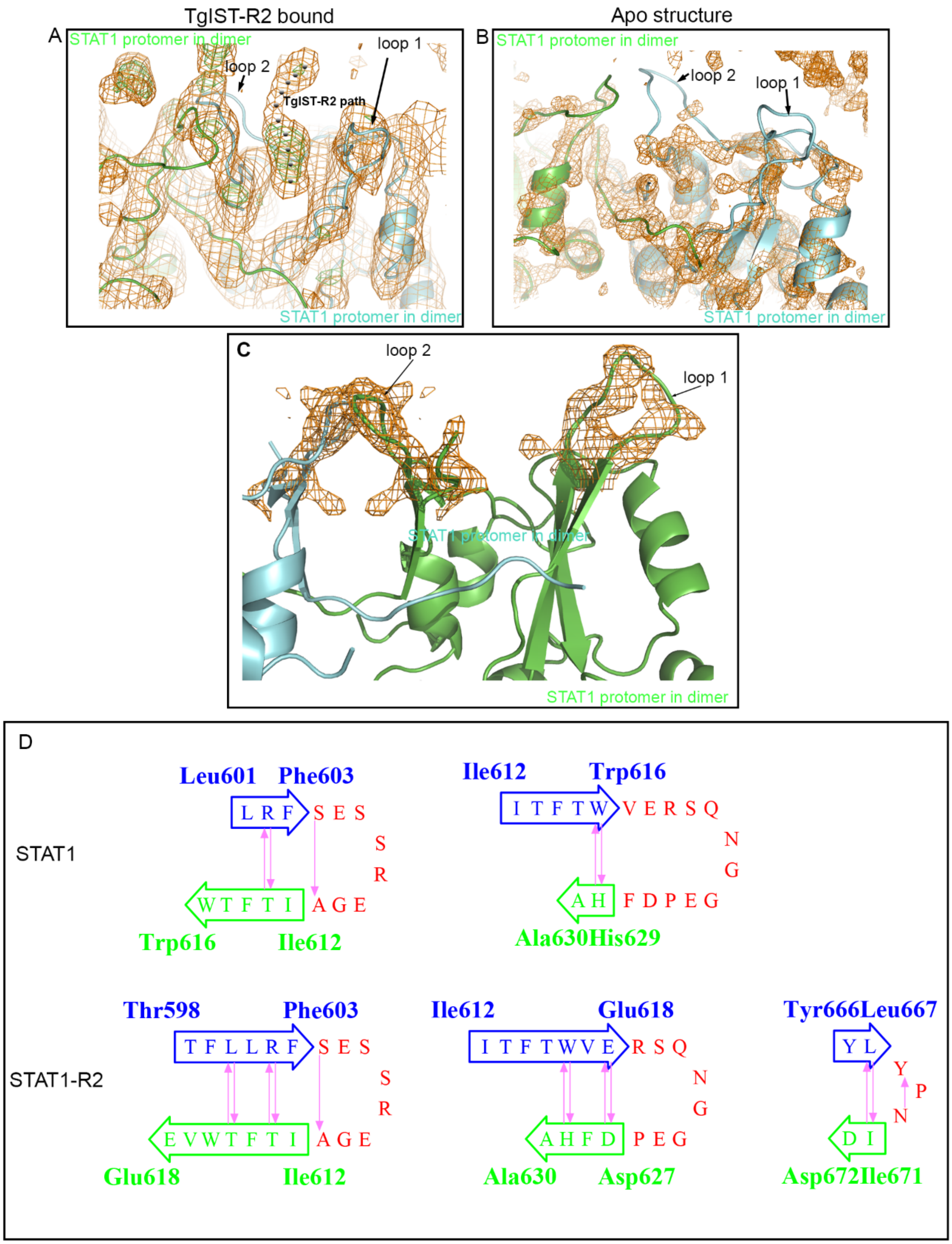

E

```
          : 365-------375-------385-------395-------405---    :
OrigSeq   : GIDRPGTTGTKGPQVRTALDVLRELTQQAKQLRLTAESSSTATASP      : OrigSeq
I-tasser  : -------------HHHHHHHHHHHHHHHHHHHSSHH----------      :I-tasser
Jnet      : -------------HHHHHHHHHHHHHHHHHHHHE------------      : Jnet
jhmm      : --------------HHHHHHHHHHHHHHHHHHHE------------      : jhmm
jpssm     : -------------HHHHHHHHHHHHHHHHHHHEEE-----------      :jpssm
AlphaFold : -----------------HHHHHHHHHHHHHHHHHH-----------      :AlphaFold
```

Supplementary Figure 8

Electron density map of loop regions in STAT1-TgIST-R2 structure and corresponding structural analysis. (a) Electron density map of TgIST-R2 bound to phosphorylated STAT1 dimer (pSTAT1d). The two proteins were purified separately and crystallized together using a molar ratio of 1:2.1 (pSTAT1d: TgIST-R2). An additional density is seen located at the top of the pSTAT1d interface formed by two flexible loops (loop 1 and loop 2). Orange mesh represents the 2Fo-Fc map contoured at 1 σ, Green mesh represents the Fo-Fc map contoured at 3 σ. Black dots represents the putative TgIST-R2 binding path between loop1 and loop 2.

(b) Electron density map of the structure of phosphorylated STAT1 dimer (pSTAT1d). Orange mesh as defined in (a) while no additional density is seen between the two loops. (c) Side view of additional densities located at the top of the pSTAT1d interface formed by two flexible loops. Orange mesh represents the 2Fo-Fc map contoured at 1.5 σ. (d) Wiring diagram of the β-sheet and hairpin analysis in the C-terminal STAT1 or STAT1-R2 structure. Figure was generated using PDBSum (http://www.ebi.ac.uk/thornton-srv/databases/cgi-bin/pdbsum/). Strand 1 (blue) and strand 2(green) form a hairpin shown in red. Main chain hydrogen bonds are indicated in purple. (e) Secondary structure prediction for TgIST-R2 using the I-TASSER server (https://zhanglab.ccmb.med.umich.edu/I-TASSER/) indicates the repeat region has a tendency to form an alpha helix. H stands for helix, C stands for coiled-coil.

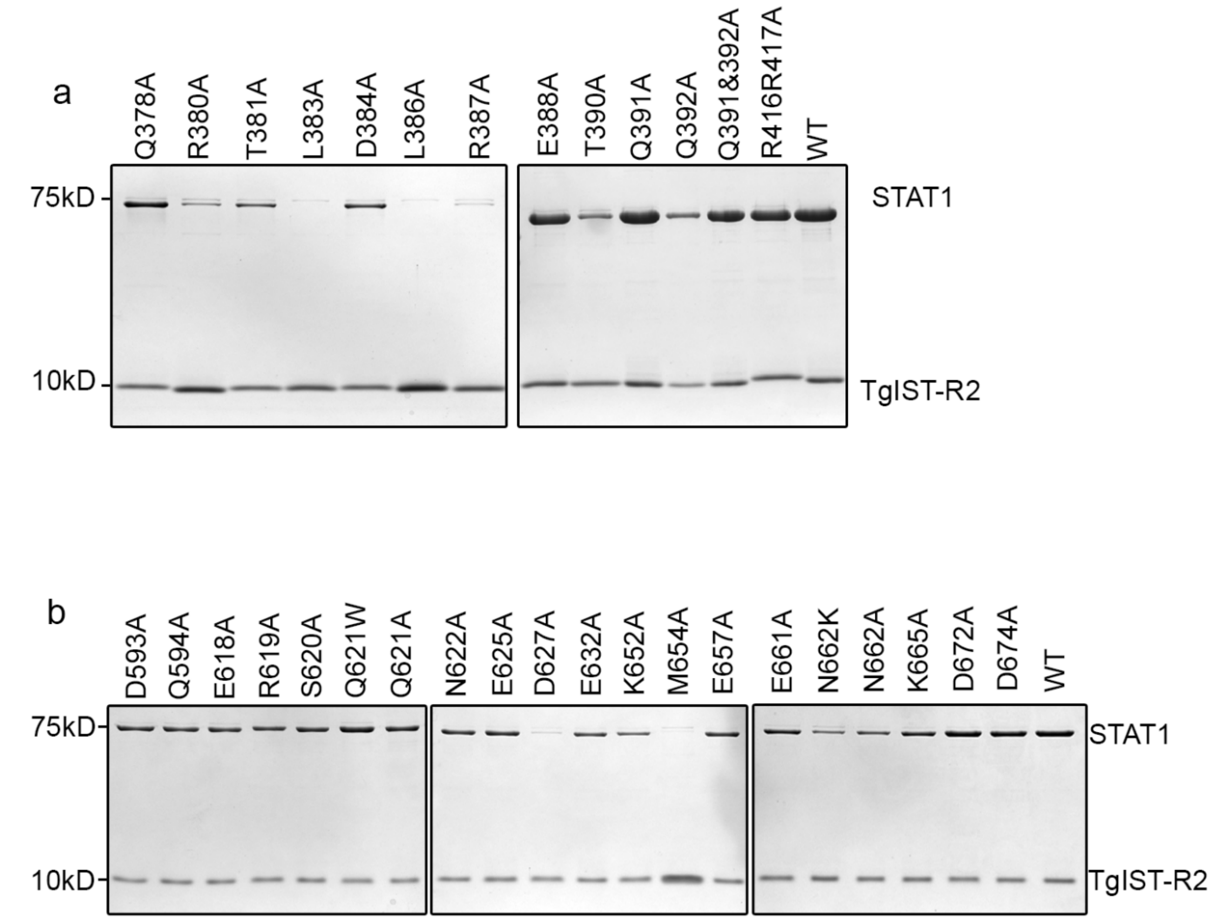

Supplementary Figure 9

Binding of His-tagged TgIST-R2 to STATcc dimer assessed by nickel affinity purification. (a) His-tagged TgIST-R2 and mutants were tested by copurification with STATcc. Mutants in TgIST are denoted above the gel. (b) His-tagged TgIST-R2 was tested by copurification with STATcc and its mutants. Mutants of STAT1cc are denoted above the gel. Eluted fractions were separated by SDS-PAGE gel and stained with Coomassie blue. One set of representative gels are shown here. Representative gel images of three independent experiments with similar results were shown here.

a

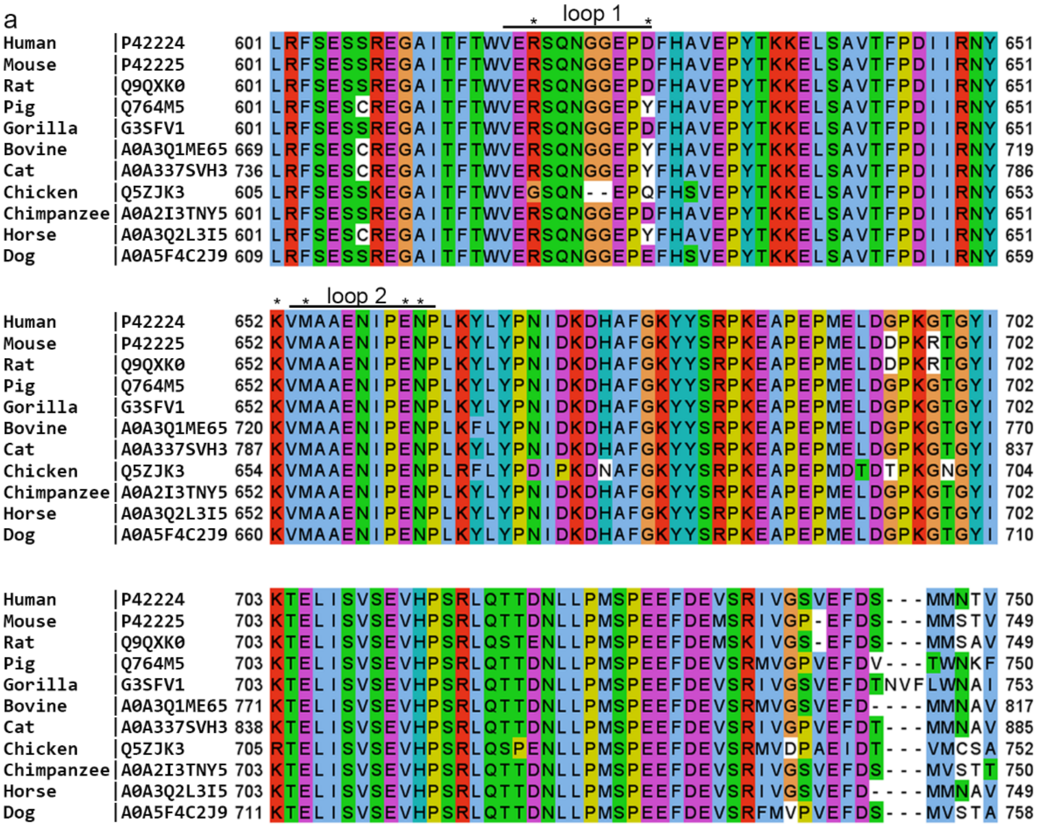

b

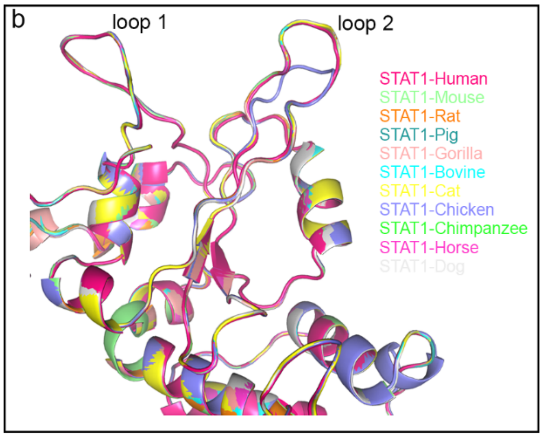

c

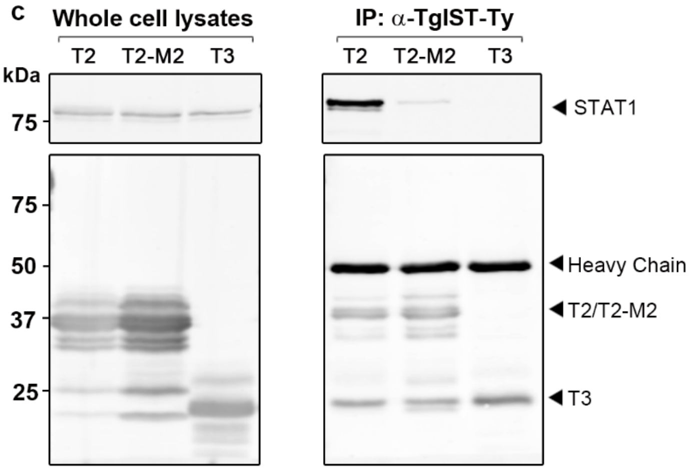

d

		Experiment 1			Experiment 2		
		HEK293T transfected with TgIST constructs:					
		TgIST-T2	TgIST-T2-M2	TgIST-T3	TgIST-T2	TgIST-T2-M2	TgIST-T3
**Scaled protein abundance**	**STAT1**	143.3	116	111.2	62.2	93.6	99.2
	**TgIST-T2**	374.7	23.8	1.4	363.3	35.9	0.7
	**CBP+p300**	82.5	171.6	105.6	57.8	147.8	83.8

e

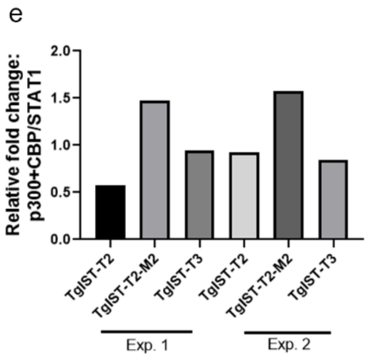

Supplementary Figure 10

The SH2 domain is conserved among STAT1 from different species and western blot analysis of proteins following immunoprecipitation of TgIST-Ty from HEK293T cells. Multiple sequence alignment of the STAT1 proteins from different species (a) and the superposition of their corresponding homology models (b). (a) STAT1 protein sequences were retrieved and imported into Jalview software (https://www.jalview.org/) and the multiple sequence alignment was performed by Muscle (https://www.ebi.ac.uk/Tools/msa/muscle/) using default settings. The sequence accession numbers of STAT1 in Uniprot database (https://www.uniprot.org/) after the species name to the left of each sequence in the alignment. Color schemes are as follows, blue: hydrophobic residues; red: positively charged residues; magenta: negatively charged residues; green: polar residues; pink: cysteines; orange: glycines; yellow: prolines; and cyan: aromatic residues. (b) Homology models of the STAT1 sequences described above were built using the Swiss-model server (https://swissmodel.expasy.org/) based on the STAT1 dimer structure (PDB: 1BF5) as the template. Models were aligned and visualized by Pymol (https://pymol.org/2/). (c) HEK293T cells were transfected with TgIST constructs expressing the mature form of TgIST (M2), a truncated form containing both repeat TgIST-T2 (T2); a mutant where the core 7 amino acids in both repeats have been replaced with alanine TgIST-T2-M2 (T2-M2); and a truncated version lack both repeats TgIST-T3, also see schematic in Fig. 1d and 3b). Cells were infected for 23 hr, then treated ± IFN-γ (100 U/mL) for additional 60 min prior to whole cell extract preparation. Membranes were incubated with corresponding primary antibodies as indicated and then IR dye-conjugated secondary antibodies. Visualization was performed using an Odyssey infrared imager. Representative blots of two independent experiments with similar results were shown here. (d) Label-free quantification by mass spectrometry of STAT1 immunoprecipitation (IP) from TgIST transfected HEK293T cells, corresponding to Fig. 7e. The peptides were quantified using the precursor abundance based on intensity. Then proteins were scaled using total peptide amount. (e) Relative fold change of CBP/p300 calculated from (d). Relative fold change was defined by using scaled abundance of CBP+p300 to divide the abundance of STAT1 in each sample.

Supplementary Table 1. Data collection and refinement statistics.

	**STAT1-linker-R2** (4 datasets merged)
**Data Collection**	
Wavelength (Å)	1.072
Resolution range (Å) [a]	46.64-2.97 (3.15-2.97)
Space group	$C\ 2\ 2\ 2_1$
Unit cell<br>(a, b, c) (Å)<br>(α, β, γ) (˚)	 59.9, 163.9, 226.9<br>90, 90, 90
Redundancy [a]	29.0 (28.4)
Unique reflections [a]	23640 (3764)
Completeness (%) [a]	100.0 (100.0)
Mean I/sigma (I) [a]	20.3 (1.9)
Wilson B-factor	79.63
R^{b}_{merge} [a]	0.176(1.961)
R^{b}_{pim} [a]	0.033 (0.372)
$CC_{1/2}$ [a]	0.999 (0.874)
**Refinement statistics**	
Reflections (work)	23585(2303)
Reflections (test)	1160 (116)
R-work	0.248
R-free	0.288
Average B_{fact} ($Å^2$)	95.33
RMS(bonds)	0.010
RMS(angles)	1.14
Ramachandran outliers (%)	0.36
MolProbity score	2.21
Clashscore all atoms	16.50

[a] Statistics for the highest-resolution shell are shown in parentheses.

[b] $R_{merge} = \Sigma_{hkl} \Sigma_i |I_{hkl,i} - \langle I_{hkl}\rangle| / \Sigma_{hkl} \Sigma_i I_{hkl,i}$ and $R_{pim} = \Sigma_{hkl} (1/(n-1))^{1/2} \Sigma_i | I_{hkl,i} - \langle I_{hkl}\rangle | / \Sigma_{hkl} \Sigma_i I_{hkl,i}$, where $I_{hkl,i}$ is the scaled intensity of the ith measurement of reflection h, k, l, $\langle I_{hkl}\rangle$ is the average intensity for that reflection, and n is the redundancy.

Supplementary Table 2. Antibodies used in this study.

REAGENT or RESOURCE	Dilution	SOURCE	IDENTIFIER
Antibodies			
Mouse monoclonal anti-Ty	1:40 for IP<br>1:1000 for blots	In house hybridoma (Bastin et al., 1996)	mAB clone BB2
Rabbit polyclonal anti-Stat1	1:100 for IP<br>1:1000 for IFA<br>1:2000 for blots	Cell Signaling Technology	Cat#9172S
Rabbit monoclonal anti-Phospho-Stat1	1:2000	Cell Signaling Technology	Cat#9167S
Rabbit monoclonal anti-MTA1	1:2000	Cell Signaling Technology	Cat#5646S
Rabbit polyclonal anti-HDAC1	1:2000	Cell Signaling Technology	Cat#34589S
Rabbit monoclonal anti-TBP	1:2000	Cell Signaling Technology	Cat#44059S
Rabbit monoclonal anti-CBP	1:1000	Cell Signaling Technology	Cat#7389S
Rabbit monoclonal anti-p300	1:1000	Cell Signaling Technology	Cat#86377S
Mouse monoclonal anti-GFP	1:1000	Thermo Fisher	Cat#A-11120
Rabbit monoclonal anti-IRF1	1:400	Cell Signaling Technology	Cat#8478S
IRDye 800CW Goat anti-mouse IgG (H+L)	1:5000	LI-COR Biosciences	Cat#925-32210
IRDye 800CW Goat anti-rabbit IgG (H+L)	1:5000	LI-COR Biosciences	Cat#925-32211
IRDye 680RD Goat anti-mouse IgG (H+L)	1:5000	LI-COR Biosciences	Cat#925-68070
IRDye 680RD Goat anti-mouse IgG (H+L)	1:5000	LI-COR Biosciences	Cat#926-68071
Alexa Fluor 488 Goat anti-mouse IgG (H+L)	1:1000	Thermo Fisher	Cat#A-11029
Alexa Fluor 568 Goat anti-mouse IgG (H+L)	1:1000	Thermo Fisher	Cat#A-11031
Alexa Fluor 647 Goat anti-rabbit IgG (H+L)	1:1000	Thermo Fisher	Cat#A-11011
PE/Cyanine7 anti-mouse I-A/I-E Antibody	1:100	Biolegend	Cat#107629
PE/Cyanine7 Rat IgG2b κ Isotype Ctrl Antibody	1:100	Biolegend	Cat#400617
TruStain FcX™ PLUS (anti-mouse CD16/32) Antibody	1:100	Biolegend	Cat#156603
