# Supplementary material for: The intrinsically disordered protein TgIST from Toxoplasma gondii inhibits STAT1 signaling by blocking cofactor recruitment
Source: Nat Commun. 2022 Jul 13;13:4047. doi: 10.1038/s41467-022-31720-7 (PMC9279507; doi:10.1038/s41467-022-31720-7)
Supplement: Supplementary file 3 — Description of Additional Supplementary Files [file 41467_2022_31720_MOESM3_ESM.pdf]

Description of Additional Supplementary Files

Supplementary Data 1. Summary of mass spectrometry analysis of human and Toxoplasma proteins immunoprecipitated with TgIST-Ty in U3A-STAT1 cells

Supplementary Data 2. Summary of mass spectrometry analysis of human and Toxoplasma proteins immunoprecipitated with TgIST-Ty in U3A-STAT1-null cells

Supplementary Data 3. Plasmids used in this study.

Supplementary Data 4. Oligonucleotides used in this study.
