# Supplementary material for: The intrinsically disordered protein TgIST from Toxoplasma gondii inhibits STAT1 signaling by blocking cofactor recruitment
Source: Nat Commun. 2022 Jul 13;13:4047. doi: 10.1038/s41467-022-31720-7 (PMC9279507; doi:10.1038/s41467-022-31720-7)
Supplement: Supplementary file 8 — Reporting Summary [file 41467_2022_31720_MOESM8_ESM.pdf]

Corresponding author(s): L. David Sibley

Last updated by author(s): Jun 15, 2022

Reporting Summary

Nature Portfolio wishes to improve the reproducibility of the work that we publish. This form provides structure for consistency and transparency in reporting. For further information on Nature Portfolio policies, see our Editorial Policies and the Editorial Policy Checklist.

Statistics

For all statistical analyses, confirm that the following items are present in the figure legend, table legend, main text, or Methods section.

n/a	Confirmed	
☐	☒	The exact sample size (*n*) for each experimental group/condition, given as a discrete number and unit of measurement
☐	☒	A statement on whether measurements were taken from distinct samples or whether the same sample was measured repeatedly
☐	☒	The statistical test(s) used AND whether they are one- or two-sided<br>*Only common tests should be described solely by name; describe more complex techniques in the Methods section.*
☒	☐	A description of all covariates tested
☐	☒	A description of any assumptions or corrections, such as tests of normality and adjustment for multiple comparisons
☐	☒	A full description of the statistical parameters including central tendency (e.g. means) or other basic estimates (e.g. regression coefficient) AND variation (e.g. standard deviation) or associated estimates of uncertainty (e.g. confidence intervals)
☐	☒	For null hypothesis testing, the test statistic (e.g. *F*, *t*, *r*) with confidence intervals, effect sizes, degrees of freedom and *P* value noted<br>*Give P values as exact values whenever suitable.*
☒	☐	For Bayesian analysis, information on the choice of priors and Markov chain Monte Carlo settings
☒	☐	For hierarchical and complex designs, identification of the appropriate level for tests and full reporting of outcomes
☒	☐	Estimates of effect sizes (e.g. Cohen's *d*, Pearson's *r*), indicating how they were calculated

Our web collection on statistics for biologists contains articles on many of the points above.

Software and code

Policy information about availability of computer code

Data collection	Gen5; Zen 3.3; Image Studio 5.2; Octet Data aquisition 10.0; ASTRA 6; Chirascan ProData software<br>Crystallographic diffraction data was collected using the standard synchrotron control software at Advanced Light Source (4.2.2) or Advanced Photon Source (19-ID)
Data analysis	Mascot; Scaffold Proteome software 5.0; Unicorn 5.20; Image Lab 6.1; Octet Data analysis 9.0; GraphPad Prism 8; CellProfiler; MUSCLE sequence alignment software; JalView 2.11; XDS; CCP4; Refmac 5; Phenix; COOT; Molprobity; PyMol 2.3;FlowJo V10; Living Image 4.7.0

For manuscripts utilizing custom algorithms or software that are central to the research but not yet described in published literature, software must be made available to editors and reviewers. We strongly encourage code deposition in a community repository (e.g. GitHub). See the Nature Portfolio guidelines for submitting code & software for further information.

Data

Policy information about availability of data

All manuscripts must include a data availability statement. This statement should provide the following information, where applicable:

- Accession codes, unique identifiers, or web links for publicly available datasets
- A description of any restrictions on data availability
- For clinical datasets or third party data, please ensure that the statement adheres to our policy

The flow cytometry data have been deposited in FlowRepository under the accession code: FR-FCM-Z5FE (flowrepository.org/id/FR-FCM-Z5FE). The crystal structure of phosphorylated STAT1 dimer complexed with repeat region from Toxoplasma protein TgIST have been deposited in Protein Data Bank with PDB ID: 8D3F(https://www.rcsb.org/structure/8D3F). The structures of STAT1 (PDB: 1BF5[https://www.rcsb.org/structure/1BF5]), monomeric STAT1 (PDB: 1YVL[https://www.rcsb.org/structure/1YVL]), STAT3 (PDB: 1BG1[https://www.rcsb.org/structure/1BG1]) and STAT6 (PDB: 5D39[https://www.rcsb.org/structure/5d39]) were downloaded from

Protein Data Bank. The proteomic data have been deposited in MassIVE under accession code MSV000089636 (https://massive.ucsd.edu/ProteoSAFe/dataset.jsp?task=35563440c033486ab6cfdd109f9bc47d). Sequences of human STAT1 were retrieved from the Uniprot database with accession number P42224; mouse, P42225(https://www.uniprot.org/uniprot/P42225); rat, Q9QXK0(https://www.uniprot.org/uniprot/ Q9QXK0); pig, Q764M5(https://www.uniprot.org/uniprot/Q764M5); gorilla, G3SFV1(https://www.uniprot.org/uniprot/ G3SFV1); bovine, A0A3Q1ME65(https://www.uniprot.org/uniprot/A0A3Q1ME65); cat, A0A337SVH3 (https://www.uniprot.org/uniprot/A0A337SVH3); chicken, Q5ZJK3(https://www.uniprot.org/uniprot/Q5ZJK3); chimpanzee, A0A2I3TNY5(https://www.uniprot.org/uniprot/A0A2I3TNY5); horse, A0A3Q2L3I5(https://www.uniprot.org/uniprot/A0A3Q2L3I5); and dog, A0A5F4C2J9(https://www.uniprot.org/uniprot/A0A5F4C2J9). Source data are provided with this paper.

Field-specific reporting

Please select the one below that is the best fit for your research. If you are not sure, read the appropriate sections before making your selection.

- [x] Life sciences
- [ ] Behavioural & social sciences
- [ ] Ecological, evolutionary & environmental sciences

For a reference copy of the document with all sections, see nature.com/documents/nr-reporting-summary-flat.pdf

Life sciences study design

All studies must disclose on these points even when the disclosure is negative.

Sample size	The number of data points collected from each sample was based on the minimum required to perform statistical comparisons (n≥3). All experiments were performed at least two independent times., Mouse numbers (n=5 in each group) were based on our previously published work and elsewhere in the literature.
Data exclusions	In IRF1 intensity quantification assays (Fig 2f and 3e), intensity value more than 2000 were excluded. These abnormal high intensity signal was caused by detachment of cells from plates resulting in a miscalculation of the intensity by software.<br>The mouse with highest and lowest signal in each group (5 mice) were removed to make the final figure.
Replication	At least two biological replicates were performed. All results were successfully replicated.
Randomization	In mouse experiments, animals were randomly allocated to each experimental group.
Blinding	We did not blind the samples. In all of the experiments, the readouts of the assays are quantitative and not subject to investigator interpretation. Variation on the outcome was addressed using replicates.

Reporting for specific materials, systems and methods

We require information from authors about some types of materials, experimental systems and methods used in many studies. Here, indicate whether each material, system or method listed is relevant to your study. If you are not sure if a list item applies to your research, read the appropriate section before selecting a response.

Materials & experimental systems

n/a	Involved in the study	
	x	Antibodies
	x	Eukaryotic cell lines
x		Palaeontology and archaeology
	x	Animals and other organisms
x		Human research participants
x		Clinical data
x		Dual use research of concern

Methods

n/a	Involved in the study	
x		ChIP-seq
	x	Flow cytometry
x		MRI-based neuroimaging

Antibodies

Antibodies used

Primary antibodies used in this study: anti-Ty (in house hybridoma, mAB clone BB2); anti-Stat1 (Cell Signaling Technology, Cat#9172S); anti-Phospho-Stat1 (Cell Signaling Technology, Cat#9167S); anti-MTA1 (Cell Signaling Technology, Cat#5646S); anti-HDAC1 (Cell Signaling Technology, Cat#34589S); anti-TBP (Cell Signaling Technology, Cat#44059S); anti-CBP (Cell Signaling Technology, Cat#7389S); anti-p300 (Cell Signaling Technology, Cat#86377S); anti-BRG1 (Santa Cruz Biotechnology Cat#sc-10768);anti-GFP (Thermo Fisher, Cat#A-11120); anti-IRF-1 (Cell Signaling Technology, Cat#8478S); PE/Cyanine7 anti-mouse I-A/I-E Antibody(BioLegend, Cat#107629); PE/Cyanine7 Rat IgG2b, κ Isotype Ctrl Antibody (BioLegend, Cat#400617); TruStain FcX™ PLUS (anti-mouse CD16/32) Antibody (BioLegend, Cat#156603)
Secondary antibodies used in this study: IRDye 800CW Goat anti-mouse IgG (H+L) (LI-COR Biosciences,Cat#925-32210);IRDye 800CW Goat anti-rabbit IgG (H+L) (LI-COR Biosciences, Cat#925-32211); IRDye 680RD Goat anti-mouse IgG (H+L) (LI-COR Biosciences,Cat#925-68070); IRDye 680RD Goat anti-mouse IgG (H+L) (LI-COR Biosciences, Cat#926-68071); Alexa Fluor 488 Goat anti-mouse IgG (H+L) (Thermo Fisher, Cat#A-11029); Alexa Fluor 568 Goat anti-mouse IgG (H+L) (Thermo Fisher, Cat#A-11031); Alexa Fluor 647 Goat anti-rabbit IgG (H+L) (Thermo Fisher, Cat#A-11011)

Validation

anti-Ty: in house hybridoma was originally obtained from: Bastin, P., Bagherzadeh, A., Matthews, K. R. & Gull, K. A novel epitope tag system to study protein targeting and organelle biogenesis in Trypanosoma brucei. Mol. Biochem. Parasitol. 77, (1996). It was validated in the lab by testing against protein standards bearing this epitope tag.
Anti-Stat1,anti-Phospho-Stat1 (58D6), anti-MTA1 (D17G10), anti-HDAC1 (D5C6U), , anti-TBP (D5C9H), anti-CBP (D6C5), anti-p300 (D8Z4E), anti-GFP (3E6), anti-IRF1 ((D5E4), anti-mouse I-A/I-E (M5/114.15.2), Rat IgG2b κ Isotype Ctrl antibody (RTK4530) and TruStain FcX™ PLUS (anti-mouse CD16/32) antibody (S17011E) were validated by the manufacturers as described in the product description.

Eukaryotic cell lines

Policy information about cell lines

Cell line source(s)

HEK293T (ATCC, CRL-11268); HeLa (ATCC, CCL-2); RAW264.7 (ATCC, TIB-71); U3A and U3A-STAT1 (Michael J. Holtzman lab)

Authentication

ATTC lines were used within a few passages from the source. The U3A and U3A-STAT1 lines were validated by Western blot for STAT1.

Mycoplasma contamination

Cultures were tested for Mycoplasma contamination using the e-Myco plus mycoplasma PCR detection kit following the manufacturer's manual (Boca Scientific).

Commonly misidentified lines
(See ICLAC register)

No commonly misidentified cell lines were used in the study.

Animals and other organisms

Policy information about studies involving animals; ARRIVE guidelines recommended for reporting animal research

Laboratory animals

C57/BL6 mice female 8-10 weeks (Charles River Lab)

Wild animals

No wild animals were used in the study.

Field-collected samples

No field collected samples were used in the study.

Ethics oversight

Animal studies were approved by the Institutional Care Committee, Division of Comparative Medicine, Washington University.

Note that full information on the approval of the study protocol must also be provided in the manuscript.

Flow Cytometry

Plots

Confirm that:

- [x] The axis labels state the marker and fluorochrome used (e.g. CD4-FITC).
- [x] The axis scales are clearly visible. Include numbers along axes only for bottom left plot of group (a 'group' is an analysis of identical markers).
- [x] All plots are contour plots with outliers or pseudocolor plots.
- [x] A numerical value for number of cells or percentage (with statistics) is provided.

Methodology

Sample preparation

Live umpermeabilized RAW264.7 cells were stained on ice with indicated antibodies.

Instrument	Sony SH800 Cell Sorter
Software	FlowJoV10
Cell population abundance	In uninfected samples, >90% of collected events gated as RAW cells by FSC/SSC. In infected samples, >50% of collected events gated as RAW cells.
Gating strategy	FSC/SSC was used to gate for viable RAW cells, SSC/GFP was used to gate infected RAW cells. Details see Supplementary Fig 6a.

☒ Tick this box to confirm that a figure exemplifying the gating strategy is provided in the Supplementary Information.
